# Diagnostic work-up of patients presenting in primary care with lower abdominal symptoms: which faecal test and triage strategy should be used?

**DOI:** 10.1186/s12916-016-0694-3

**Published:** 2016-09-26

**Authors:** Callum G. Fraser

**Affiliations:** Centre for Research into Cancer Prevention and Screening, University of Dundee, Ninewells Hospital and Medical School, Dundee, DD1 9SY Scotland UK

**Keywords:** Colorectal cancer, Faecal calprotectin, Faecal haemoglobin, Faecal immunochemical test, Point-of-care, Primary care, Significant colorectal disease

## Abstract

Bowel endoscopy referrals from primary care have increased steadily over recent years. However, most patients do not have significant colorectal disease (SCD). Therefore, strategies to select those who would benefit most from endoscopy are of current interest. A recent study developed a multivariable diagnostic model for SCD with routine clinical information, extended with quantitative faecal calprotectin (f-C) point-of-care (POC) testing and/or qualitative POC faecal immunochemical test (FIT) for haemoglobin (f-Hb) results. This study used POC tests for both f-C and f-Hb; however, POC tests have many disadvantages and there are several reasons why quantitative measurements of f-Hb are advantageous. Quantitative faecal immunochemical tests have been used very successfully in triage of patients presenting in primary care as a rule-out test. Studies have compared f-C and f-Hb in this clinical context and consider that f-C is not required in diagnosis. A single quantitative f-Hb result, without any clinical information, could be sufficient to decide whom to refer for endoscopy and, because of the significant overlap of symptoms in those with and without SCD, could be the primary investigation performed.

Please see related article: http://bmcmedicine.biomedcentral.com/articles/10.1186/s12916-016-0684-5.

## Background

The number or requests for lower gastrointestinal endoscopy has continued to increase in recent years, partly due to the success of colorectal cancer (CRC) screening programmes and the surveillance of greater number of patients with known and treated disease, but mainly due to referrals from primary care to investigate patients with lower abdominal symptoms. As documented by Elias et al. [1], the majority of these patients do not have significant colorectal disease (SCD) and are unnecessarily exposed to an unpleasant and invasive procedure that carries a small, but realistic, risk of complications. Therefore, and considering the very limited endoscopy resources in several countries, strategies to select those patients who would benefit most from endoscopy are of considerable interest, as exemplified by the work under way by the National Institute for Health and Care Excellence (NICE) on quantitative faecal immunochemical tests (FITs) to assess symptomatic people presenting in primary care who are at low risk of CRC [2].

### Faecal tests and triage strategies in patients with lower abdominal symptoms

Elias et al. [1] used data from a prospective diagnostic study in patients from primary care practices with persistent lower abdominal symptoms referred for endoscopy to develop a multivariable diagnostic model for SCD with routine clinical information, which was extended with quantitative faecal calprotectin (f-C) point-of care (POC) test data and/or qualitative POC FIT for haemoglobin (f-Hb) results. SCD was defined as CRC, inflammatory bowel disease, diverticulitis or advanced adenoma (simplistically, as those above 1 cm in diameter) detected at endoscopy. Of 810 patients, 141 (17.4 %) had SCD, supporting the “rule of sixths” that one-sixth of patients presenting in primary care have SCD, two-sixths have other, less significant bowel disorders, and three-sixths have a normal colon on endoscopy [3].

This study used POC tests for both f-C and f-Hb, the rationale being that these can be easily executed at the time and place of patient care. However, POC tests have several disadvantages – currently available POC FITs have considerable differences with regard to their analytical detection limit [4]. Therefore, the conclusions of Elias et al. [1] may not be applicable to other POC FITs with varying analytical sensitivity and specificity. Moreover, faecal specimen collection is problematic since f-Hb is unstable, as confirmed by Elias et al. [1], requiring the use of simple and hygienic specimen collection devices rather than the traditional “stool pots”. Further, the colour development on immunochromatographic POC tests is very dynamic and early or late reading will lead to false negative or false positive results, respectively. Therefore, the results are not easy to interpret, especially when borderline positive, unless performed following adequate training and in good light, and preferably by those with good visual acuity. Thus, there are several reasons why high-quality quantitative measurements of f-Hb performed in ISO 15189 accredited laboratories are much preferred. As discussed by Elias et al. [1], these FITs have now been used very successfully in triage of symptomatic patients presenting in primary care and allow improved diagnostic information to be gained. However, it might be that use of central laboratory-based tests would cause some, albeit small, delay in diagnosis and, more importantly, some drop out and loss of patients from pathways after presentation. It would be of interest to undertake an objective comparison of the efficiency and effectiveness of POC as compared to central laboratory testing.

Elias et al. [1] demonstrated that a diagnostic model with routine clinical data discriminated between patients with and without SCD with an area under the receiver operating characteristic curve (AUC) of 0.741. This AUC increased to 0.763 when adding the f-C and to 0.831 when adding the f-Hb, and to 0.837 upon combined extension; 30.4 % of the patients tested negative based on this combined POC test extended model, with a 96.4 % negative predictive value (NPV). This high NPV indicates that this approach is a good rule-out test for SCD. However, excluding the f-C from this model still yielded 96.0 % NPV. It was concluded that a diagnostic strategy with routine clinical data and f-Hb alone may safely rule out SCD and prevent unnecessary endoscopy referral in approximately one-third of patients. Interestingly, there are other studies which have compared f-C and f-Hb in this clinical context [5, 6], and they too consider that f-C is not required. Patients with SCD have higher concentrations of f-Hb, since f-Hb is related to the severity of colorectal disease [7], and there is now much evidence that f-Hb alone has huge potential for use in risk stratifying of symptomatic patients. Indeed, there is growing evidence that f-Hb is more useful than f-C in the assessment and monitoring of ulcerative colitis [8]. Thus, perhaps in the near future, when quantitative f-Hb becomes more widely available for triage of symptomatic patients, f-C will be used mainly in the monitoring of patients with known inflammatory bowel disease rather than in the diagnostic setting. Further research is needed to compare f-C and f-Hb in a wide range of gastrointestinal disorders.

There is much evidence that a single f-Hb result could be sufficient to decide whom to refer for endoscopy [9]. Indeed, Elias et al. [1] state that their results also underscore that a positive f-Hb already implies the need for referral and admit that the clinical data do not add much. However, it was suggested that such data are informative when the f-Hb result is negative. Moreover, it was alleged that, in daily clinical practice, and certainly in primary care, it is rare that primary healthcare professionals would immediately request tests in patients presenting with symptoms and signs of SCD without even considering any other pre-test diagnostic information from history taking and physical examination. It was argued that the diagnostic process in primary care is sequential, starting with history taking and physical examination, with follow-up testing only in cases where these provide indications that support additional testing. Thus, in order to ensure adherence to primary care practice, Elias et al. [1] explicitly initially evaluated the diagnostic value of history taking, physical examination, and simple blood testing and, subsequently, the added value of the POC f-Hb, rather than in reverse. However, the introduction to the work of Elias et al. [1] and to this commentary both indicate that, although lower abdominal symptoms are very common presenting complaints, SCD is rarer. Because of the significant overlap of symptoms in those with and without SCD, it could be argued that, because f-Hb is such a good rule-out test for SCD, this should be the primary and first investigation to be performed.

## Conclusions

Deciding which patients presenting with lower abdominal symptoms will benefit most from endoscopy is problematic. Combined faecal biomarkers, such as f-C and f-Hb, have been advocated, but f-Hb alone has advantages. Qualitative estimates of f-Hb also provide more information than qualitative f-Hb POC tests. Simple and rapid diagnostic pathways are also advantageous. Thus, a possible strategy would be to collect a sample for qualitative measure of f-Hb from all patients presenting with lower abdominal symptoms in primary care. Following this, senior endoscopy staff could assess, taking the f-Hb results and symptoms into account, whether there is a need for the patient to undergo endoscopy. Nevertheless, there are, of course, other alternative strategies and it is hoped that future research will explore the best applications of f-Hb in the triage of the symptomatic, although these might be highly dependent on local circumstances and approaches.
