# Erratum to: Diagnostic work-up of patients presenting in primary care with lower abdominal symptoms: which faecal test and triage strategy should be used?

**DOI:** 10.1186/s12916-016-0713-4

**Published:** 2016-10-12

**Authors:** Callum G. Fraser

**Affiliations:** Centre for Research into Cancer Prevention and Screening, University of Dundee, Ninewells Hospital and Medical School, Dundee, DD1 9SY Scotland UK

## Erratum

After publication of the original article [1], it came to the author’s attention that there were two errors in the Conclusions section. ‘Quantitative’ was mistakenly written as ‘qualitative’ in the following part of the section:

“**Quantitative** estimates of f-Hb also provide more information than qualitative f-Hb POC tests. Simple and rapid diagnostic pathways are also advantageous. Thus, a possible strategy would be to collect a sample for **quantitative** measure of f-Hb from all patients presenting with lower abdominal symptoms in primary care.”
